# Health management program: factors influencing completion of therapy with high-dose interferon alfa-2b for high-risk melanoma

**DOI:** 10.3747/co.2008.200

**Published:** 2008-01

**Authors:** N. Levesque, K. Mitchinson, D. Lawrie, L. Fedorak, D. MacDonald, C. Normand, J.F. Pouliot

**Affiliations:** * Hamilton Regional Cancer Centre, Hamilton, ON; † Royal Victoria Hospital, Barrie, ON; ‡ Sunnybrook Regional Cancer Centre, Toronto, ON; § Cross Cancer Institute, Edmonton, AB; || Mississauga, ON; # Centre Hospitalier des Vallées de l’Outaouais, Gatineau, QC; ** Schering-Plough Canada, Pointe-Claire, QC

**Keywords:** Melanoma, interferon alfa-2b, clinical trial, adherence

## Abstract

The goal of the 1-year observational, multicentre, open-label study reported here was to identify factors influencing adherence to high-dose interferon alfa-2b adjuvant therapy in patients at high risk of recurrence following surgical excision of malignant melanoma. The study was carried out in 23 tertiary-care centres across Canada.

The 225 patients enrolled in the study all had malignant melanoma that was surgically excised and that required adjuvant treatment with interferon alfa-2b. Of these patients, 64% were men. Mean age was 51.7 years. All patients received interferon alfa-2b treatment during a 4-week induction phase (20 MU/m^2^ intravenously 5 days per week) followed by a 48-week maintenance phase (10 MU/m^2^ subcutaneously 3 days per week).

Oncology nurses reviewed side-effect management with the patients before the induction and maintenance phases. Patients were provided with daily diaries, comprehensive educational materials, and ongoing nursing support. Data on side effects and discontinuations were obtained from patient interviews and diaries.

The main outcome measurements were related to treatment discontinuation: rate, timing, reason, and prevention. Of the 225 patients, 75 (33.3%) discontinued interferon during the induction phase, and 58 (25.8%) discontinued during the maintenance phase. The main reasons for discontinuation were adverse events (58%) and disease progression (26%). Patients with a daily fluid intake greater than 1.5 L were more likely to complete therapy than were those with an intake less than 1.5 L (64% vs. 36%, *p* < 0.0001).

Of 225 patients enrolled in the interferon alfa-2b health management program, 41% completed the 1-year treatment course. Higher fluid intake (>1.5 L daily) was associated with increased adherence to therapy.

## 1. INTRODUCTION

In Canada, an estimated 4500 people are diagnosed with melanoma annually. Melanoma ranks as the 7th most frequently diagnosed cancer among Canadian men and the 8th among Canadian women, with an age-standardized incidence of 9–23 cases per 100,000 1. Malignant melanoma accounts for an estimated 4% of skin cancer cases, but it represents more than 79% of all skin cancer deaths 2. The incidence of melanoma is reported to be increasing at a rate of 2% annually 3.

Interferons are cytokines with a range of biologic effects, including antiproliferative and antiviral activity. In the clinical setting, interferon alfa-2b (Intron A: Schering–Plough, Kenilworth, NJ, U.S.A.) is indicated for the treatment of malignant melanoma, hairy cell leukemia, follicular lymphoma, chronic myelogenous leukemia, multiple myeloma, condylomata acuminata, chronic hepatitis b and c, and aids-related Kaposi sarcoma 4. Interferon alfa-2b is the only adjuvant therapy that has been shown to increase survival in high-risk melanoma patients; it is currently being investigated as neoadjuvant therapy 5,6.

The Eastern Cooperative Oncology Group (ecog) trial 1684 randomized 287 patients with high-risk resected cutaneous melanoma to a maximum tolerated dose of interferon alfa-2b 20 MU/m^2^ intravenously 5 days per week for 4 weeks, followed by 10 MU/m^2^ subcutaneously 3 days per week for 48 weeks, or to observation 7. As compared with observation, adjuvant treatment was associated with significant prolongation of disease-free survival to 1.7 years from 1 year and an increase in median overall survival to 3.8 years from 2.8 years during the median follow-up period of 6.9 years. Additional cooperative group studies also demonstrated significant improvements in relapse-free survival (two studies) and overall survival (one study) with high-dose interferon alfa-2b 5,8.

**FIGURE 1:**
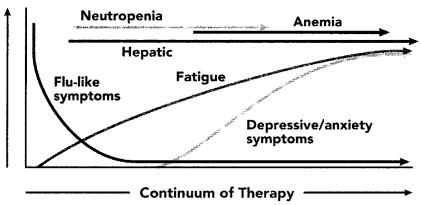
Time of occurrence of interferon-related side effects during the course of treatment (adapted, with permission, from Kirkwood et al., 2002 9)

However, treatment with recombinant interferon alfa-2b is associated with significant side effects and is often poorly tolerated by patients. Medication-related side effects can occur throughout the course of treatment (Figure 1), possibly leading to early discontinuation of therapy or suboptimal drug exposure. The most common drug-related adverse effects are chronic fatigue, myelosuppression, elevated liver enzymes, and neurologic symptoms. Depression or other psychiatric symptoms may also affect quality of life and may further contribute to poor adherence to the regimen 9–11. Dose modifications are often required, although delivery of 80% or more of the scheduled dose can still be achieved in most patients 7.

Maintaining an adequate dose and duration of interferon alfa-2b therapy appears to be crucial to success of the therapy. Recent data have suggested that the treatment benefits of adjuvant high-dose in-terferon alfa-2b may be dose-dependent 12. Continuous treatment at dosages greater than 10 MU daily has also been reported to be required to achieve a clinical benefit 13.

Patients have been shown to value recurrence-free survival more highly than severe interferon toxicity 14. However, treatment discontinuation is a frequent occurrence, and little is known about patient attitudes and behaviours that might influence early cessation of treatment 15.

Ongoing nursing support appears to be one factor that is crucial to the success of interferon alfa-2b therapy for melanoma. An analysis of nursing issues arising from the ecog 1684 trial identified the important roles of accurate nursing assessments and ongoing interventions in maintaining patients on therapy. With nursing intervention, 74% of non-relapsing patients were able to complete the 1-year course of interferon alfa-2b treatment 16.

To investigate possible patient factors influencing treatment discontinuation, the Intron A Health Management Program was initiated as a structured program of nurse counselling and support. It was hypothesized that close monitoring and frequent follow-ups would help nurses to identify factors that might be contributing to early discontinuation of interferon alfa-2b regimen by patients during the 52 weeks of therapy.

## 2. PATIENTS AND METHODS

### 2.1 Study Objectives

The Intron A Health Management Program was initiated to identify factors influencing adherence to high-dose interferon alfa-2b adjuvant therapy in patients at high risk of recurrence following surgical resection of their malignant melanoma.

### 2.2 Study Design

Consecutive patients with malignant melanoma who met the inclusion criteria were eligible for enrolment in the 1-year open-label study.

### 2.3 Sample and Setting

Adult patients who had received prior surgical treatment for malignant melanoma and who were candidates for adjuvant interferon treatment were eligible for inclusion in the study. Subjects were enrolled at 23 Canadian tertiary-care centres. Most of the patients came from the Canadian provinces of Ontario (48%), Alberta (18%), Quebec (11.0%), and British Columbia (10%). All subjects were enrolled between March 2001 and September 2004.

### 2.4 Treatment

All subjects received interferon alfa-2b (Intron A). Adjuvant treatment was initiated within 56 days after surgery. The scheduled dose of interferon alfa-2b was 20 MU/m^2^ administered intravenously 5 days per week during a 4-week induction phase, followed by 10 MU/m^2^ administered subcutaneously 3 times per week during a 48-week maintenance phase.

### 2.5 Evaluations

At study entry, a physician reviewed the course of the interferon treatment, the potential adverse events, side effects management, and reimbursement issues with every patient. Patients were provided with a contact number to report medication side effects. Oncology nurses provided patients with educational materials, including a video.

At the start of the 48-week maintenance phase (week 5), patients were asked if they had reviewed the educational materials. In preparation for self-administration at home, the physician instructed patients on the proper use of interferon alfa-2b and either administered the first injection or observed the patient’s self-injection. Nurses provided patients with a daily diary and instructed them to record the site of injection, use of concomitant medications, and daily fluid intake.

The injection sites, treatment adherence, and fluid intake were recorded from patient diary entries and from patient contacts during scheduled visits or by telephone. During the induction phase, 4 follow-up visits were scheduled, and during the maintenance phase, 11 visits. During the maintenance phase, patient contacts to capture data and provide support were performed mainly by the oncology nurses. The chief research variables were the drug discontinuation rate, timing of discontinuation, reason for discontinuation, and variables that affected compliance with therapy.

## 3. RESULTS

### 3.1 Demographics

A total of 225 patients with malignant melanoma requiring adjuvant treatment with interferon alfa-2b were analyzed. Almost two thirds of the study population (64%) were men. Mean age at study entry was 51.7 years. Mean body weight was 89.8 kg for men and 73.6 kg for women.

### 3.2 Medication Use

During self-administration of the interferon alfa-2b in the 48-week maintenance phase, patients received a mean dose of 9.2 MU/m^2^ per injection. Most patients (94.8%) used a multidose pen format rather than a premixed solution (4.0%) or lyophilized powder (1.1%). The preferred injection sites as recorded in the patient diaries were the abdomen (36.0%), right leg (22.5%), left leg (21.7%), left arm (9.7%), and right arm (9.1%).

### 3.3 Contacts with Health Professionals

Patients had a mean of 9.8 contacts, defined as a clinic visit or telephone follow-up, with a physician or nurse. Most contacts were clinic visits (86.4% vs. 13.6% telephone calls). Considering all contacts, 64.1% of patients completed their diary every day, and 91.5% were adherent with the treatment regimen.

### 3.4 Fluid Intake

The average quantity of fluid consumed daily by the patients was determined to be 1.70 L. Two thirds of the patients consumed more than 1.5 L of fluids daily. The most common beverages were water (54.2%), juice (21.6%), tea (6.2%), and milk (4.0%). Abstention from alcohol consumption was reported by 96.4% of the patients.

**FIGURE 2:**
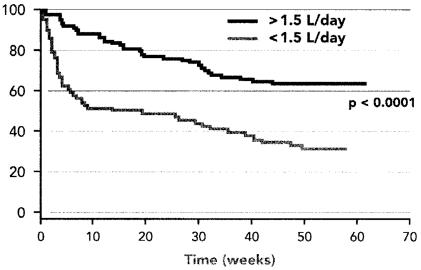
Treatment adherence among patients with high (>1.5 L) and low (<1.5 L) daily fluid intake during a 1-year course of interferon alfa-2b.

The Health Management Program identified that patients with a daily fluid intake greater than 1.5 L were more likely to complete therapy than were those with a daily fluid intake less than 1.5 L (64% vs. 36%, *p* < 0.0001, Figure 2). The beneficial impact of hydration was observed during the induction and maintenance phases alike. Patients with a daily fluid intake greater than 1.5 L stayed on therapy longer than did those with a fluid intake less than 1.5 L (mean: 39.5 weeks vs. 25.6 weeks; *p* < 0.0001).

### 3.5 Therapy Discontinuation

**FIGURE 3:**
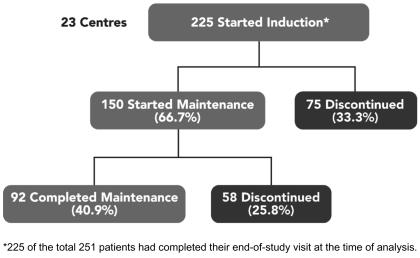
Discontinuations occurring during the induction and maintenance phases of a 1-year course of treatment with interferon alfa-2b.

During the 52-week treatment period, 59% of patients discontinued therapy: 75 of 225 patients (33.3%) discontinued during the induction phase, and 58 (25.8%) discontinued during the maintenance phase (Figure 3). The mean duration of treatment for patients who discontinued therapy was 18.3 weeks. If discontinuations attributable to disease progression or death are omitted, 51% of patients completed the full year of therapy: 25% discontinued during the induction phase, and 30% of the remainder did not complete maintenance therapy.

The most common reasons for discontinuation at any time during therapy (Table I) were adverse events (58%) and treatment failure (26%). During the study, 4 disease-related deaths (2.7%) occurred, and 3 patients (2.0%) were lost to follow-up.

The adverse events most frequently cited as leading to discontinuation of therapy were asthenia, nausea and vomiting, liver function abnormalities, anorexia, depression, and myalgia.

### 3.6 Continuation

Although the discontinuation rate was high, one third of discontinuations occurred during the 4-week induction phase. Thereafter, with ongoing nursing support, 92 of the 150 patients who started the 48-week maintenance phase (61.3%) were able to complete the course of treatment.

### 3.7 Non-adherence

Of the 92 patients who completed 1 year of therapy, 93% were adherent to the regimen more than 80% of the time. Men were more likely to be adherent than were women (*p* = 0.043). The first episode of non-adherence occurred more often during the induction phase (72.9%) than during the maintenance phase (33.6%). Overall, patients completed a mean of 11.6 weeks of treatment before their first episode of non-adherence. During the induction phase, non-adherence most commonly occurred in weeks 3 and 4; during the maintenance phase, it occurred most often in weeks 24 and 25.

### 3.8 Dose Change

Of the 225 study patients, 142 (63.1%) required a change in their dose of interferon alfa-2b. Dose changes most commonly occurred during the induction phase (78.2% vs. 21.8% in the maintenance phase). The adverse events that most commonly led to a change in dose were liver enzyme changes (43% of patients), neutropenia (21%), asthenia (10%), anorexia (6%), and nausea (4%). A dose change during the induction phase improved the patients’ likelihood of completing the course of therapy (*p* < 0.01).

### 3.9 Adverse Events

Of all 225 patients, 207 (92%) reported at least 1 adverse event. Among patients reporting an adverse event, the mean number of events was 4.5 per patient. The adverse events that most frequently led to therapy discontinuation (Table II) were asthenia (20%), nausea (13%), liver function abnormalities (11%), and anorexia (9%). Liver function abnormalities occurred in 2.3% of the patients, and elevations in hepatic enzymes, in 2.2%.

At least 1 serious adverse event possibly related to the study medication occurred in 56 patients (24.9%). Serious adverse events included neutropenia (*n* = 9), elevation in hepatic enzymes (alanine aminotransferase, gamma-glutamyl transferase, serum glutamic oxaloacetic transaminase; *n* = 6), hematologic abnormalities (*n* = 5), and depression (*n* = 2).

## 4. CONCLUSIONS

Interferons used in the treatment of various cancers, including malignant melanoma, are well known to cause a range of side effects that can compromise the success of treatment. In the present study, excluding patients who progressed or died, only 49% of patients completed 1 year of therapy. That rate is significantly lower than the completion rates reported in earlier studies by Kirkwood *et al.* (74%–90%) 7,8, highlighting the difference between controlled clinical trials and the clinical practice setting. However, oncology nurses can play a key role in patient follow-up and support and can have a significant effect on patient adherence to therapy.

The Health Management Program of nurse counselling and support observed that patients with greater daily fluid intake (>1.5 L) were significantly more likely to continue on a 52-week regimen of interferon alfa-2b therapy. Adequate hydration was also significantly correlated with a longer average duration of therapy. That finding may be partly attributable to the fact that patients who feel well may be more likely to drink more fluids. A decline in fluid intake could predict the emergence of side effects in some patients, although that hypothesis requires further study. However, the magnitude of the difference in compliance observed between patients with greater and lesser fluid intakes suggests that fluid intake was unlikely to be only a surrogate for well-being. A follow-up study, the Health Management Program II, will further investigate the correlation between hydration (oral and intravenous) and compliance. Other factors that may potentially influence compliance, such as concomitant medication use and social supports, will also be examined.

The Health Management Program reported here also demonstrated that dose adjustments improved the likelihood that patients would continue on interferon alfa-2b therapy, a finding that has been reported previously 17.

For patients entering the maintenance phase, most therapy discontinuations were the result of adverse events. However, many adverse events might be managed successfully with adequate hydration, dose reductions, and nursing or other interventions.

In conclusion, the Melanoma Health Management Program of nurse counselling and support highlighted the need to emphasize the importance of adequate hydration and to devise, in consultation with patients, effective strategies to manage adverse events to ensure that patients maximized their adherence to therapy.

## Figures and Tables

**TABLE I:** Reasons for therapy discontinuation during a 1-year course of treatment with interferon alfa-2b in patients (*n* = 225) with resected malignant melanoma

Reason	(%)
Adverse events	58
Disease progression	26
Non-compliance	4
Death	3
Lost to follow-up	2
Other	7

**TABLE II:** Adverse events leading to therapy discontinuation in patients (*n* = 134) with resected malignant melanoma taking interferon alfa-2b

Adverse event	(%)
Asthenia	20
Nausea	13
Liver function abnormalities	11
Anorexia	9
Headache	7
Depression	7
Malaise	4
Neutropenia	4
Other	25

## References

[1] Canadian Cancer Society and the National Cancer Institute of Canada (2006). Canadian Cancer Statistics 2006.

[2] Rataj D, Jankowiak B, Krajewska–Kulak E (2005). Quality-of-life evaluation in an interferon therapy after radical surgery in cutaneous melanoma patients. Cancer Nurs.

[3] United States, National Institutes of Health, National Cancer Institute, Surveillance, Epidemiology, and End Results (seer) database, 1975–2003. Trends (annual percent change) of seer incidence rates [Web resource].

[4] Kirkwood JM, Ernstoff MS (1990). Role of interferons in the therapy of melanoma. J Invest Dermatol.

[5] Kirkwood JM, Ibrahim JG, Sosman JA (2001). High-dose interferon alfa-2b significantly prolongs relapse-free and overall survival compared with the GM2-KLH/QS-21 vaccine in patients with resected stage IIB-III melanoma: results of Intergroup trial E1694/S9512/C509801. J Clin Oncol.

[6] Moschos SJ, Edington HD, Land SR, Rao UN, Shipe–Spotloe J, Kirkwood JM (2006). Neoadjuvant treatment of regional stage IIIB melanoma with high-dose interferon alfa-2b induces objective tumor regression in association with modulation of tumor infiltrating host cellular immune responses. J Clin Oncol.

[7] Kirkwood JM, Strawderman MH, Ernstoff MS, Smith TJ, Borden EC, Blum RH (1996). Interferon alfa-2b adjuvant therapy of high-risk resected cutaneous melanoma: the Eastern Cooperative Oncology Group Trial est 1684. J Clin Oncol.

[8] Kirkwood JM, Ibrahim JG, Sondak VK (2000). High- and low-dose interferon alfa-2b in high-risk melanoma: first analysis of Intergroup trial E1690/S9111/C9190. J Clin Oncol.

[9] Kirkwood JM, Bender C, Agarwala S (2002). Mechanisms and management of toxicities associated with high-dose interferon alfa-2b therapy. J Clin Oncol.

[10] Trask PC, Paterson AG, Esper P, Pau J, Redman B (2004). Longitudinal course of depression, fatigue, and quality of life in patients with high risk melanoma receiving adjuvant interferon. Psychooncology.

[11] Greenberg DB, Jonasch E, Gadd MA (2000). Adjuvant therapy of melanoma with interferon-alpha-2b is associated with mania and bipolar syndromes. Cancer.

[12] Fluck M, Kamanabrou D, Lippold A, Reitz M, Atzpodien J (2005). Dose-dependent treatment benefit in high-risk melanoma patients receiving adjuvant high-dose interferon alfa-2b. Cancer Biother Radiopharm.

[13] Kirkwood JM, Ernstoff MS (1986). Potential applications of the interferons in oncology: lessons drawn from studies of human melanoma. Semin Oncol.

[14] Kilbridge KL, Weeks JC, Sober AJ (2001). Patient preferences for adjuvant interferon alfa-2b treatment. J Clin Oncol.

[15] Kiley KE, Gale DM (1998). Nursing management of patients with malignant melanoma receiving adjuvant alpha interferon-2b. Clin J Oncol Nurs.

[16] Donnelly S (1998). Patient management strategies for interferon alfa-2b as adjuvant therapy of high-risk melanoma. Oncol Nurs Forum.

[17] Romanini A, Sarti S, Murr R (2005). Toxicity of adjuvant treatment with high-dose interferon alpha 2b (hd-ifn) in high-risk stage III melanoma patients (pts): the Italian experience [abstract]. Proc Am Soc Clin Oncol.

